# A Novel MCPH1 Isoform Complements the Defective Chromosome Condensation of Human MCPH1-Deficient Cells

**DOI:** 10.1371/journal.pone.0040387

**Published:** 2012-08-30

**Authors:** Ioannis Gavvovidis, Isabell Rost, Marc Trimborn, Frank J. Kaiser, Josephine Purps, Constanze Wiek, Helmut Hanenberg, Heidemarie Neitzel, Detlev Schindler

**Affiliations:** 1 Department of Human Genetics, University of Würzburg, Würzburg, Germany; 2 Max-Delbrück-Center for Molecular Medicine, Berlin, Germany; 3 Department of Medical Genetics, Charité - Universitaetsmedizin Berlin, Berlin, Germany; 4 Institut für Humangenetik, Universität zu Lübeck, Lübeck, Germany; 5 Department of Human Genetics, Charité - Universitaetsmedizin Berlin, Berlin, Germany; 6 Department of Otorhinolaryngology and Head & Neck Surgery, Heinrich Heine University School of Medicine, Düsseldorf, Germany; 7 Department of Pediatrics, Riley Hospital for Children, Indiana University School of Medicine, Indianapolis, Indiana, United States of America; 8 Department of Medical & Molecular Genetics, Indiana University School of Medicine, Indianapolis, Indiana, United States of America; National Institute on Aging, United States of America

## Abstract

Biallelic mutations in *MCPH1* cause primary microcephaly (MCPH) with the cellular phenotype of defective chromosome condensation. *MCPH1* encodes a multifunctional protein that notably is involved in brain development, regulation of chromosome condensation, and DNA damage response. In the present studies, we detected that *MCPH1* encodes several distinct transcripts, including two major forms: full-length MCPH1 (MCPH1-FL) and a second transcript lacking the six 3′ exons (MCPH1Δe9–14). Both variants show comparable tissue-specific expression patterns, demonstrate nuclear localization that is mediated independently via separate NLS motifs, and are more abundant in certain fetal than adult organs. In addition, the expression of either isoform complements the chromosome condensation defect found in genetically MCPH1-deficient or MCPH1 siRNA-depleted cells, demonstrating a redundancy of both MCPH1 isoforms for the regulation of chromosome condensation. Strikingly however, both transcripts are regulated antagonistically during cell-cycle progression and there are functional differences between the isoforms with regard to the DNA damage response; MCPH1-FL localizes to phosphorylated H2AX repair foci following ionizing irradiation, while MCPH1Δe9–14 was evenly distributed in the nucleus. In summary, our results demonstrate here that *MCPH1* encodes different isoforms that are differentially regulated at the transcript level and have different functions at the protein level.

## Introduction

Primary autosomal recessive microcephaly (MCPH, OMIM 606858) is a non-syndromic neurodevelopmental disorder in which the cerebral cortex volume is significantly reduced. The head circumference of MCPH patients is at least three standard deviations below that of unaffected individuals of the same age and sex [1]. MCPH patients exhibit grossly normal brain architecture: however, they also exhibit simplified gyral patterns [2]. Affected individuals are mentally retarded but lack significant neurological deficits.

**Figure 1:**
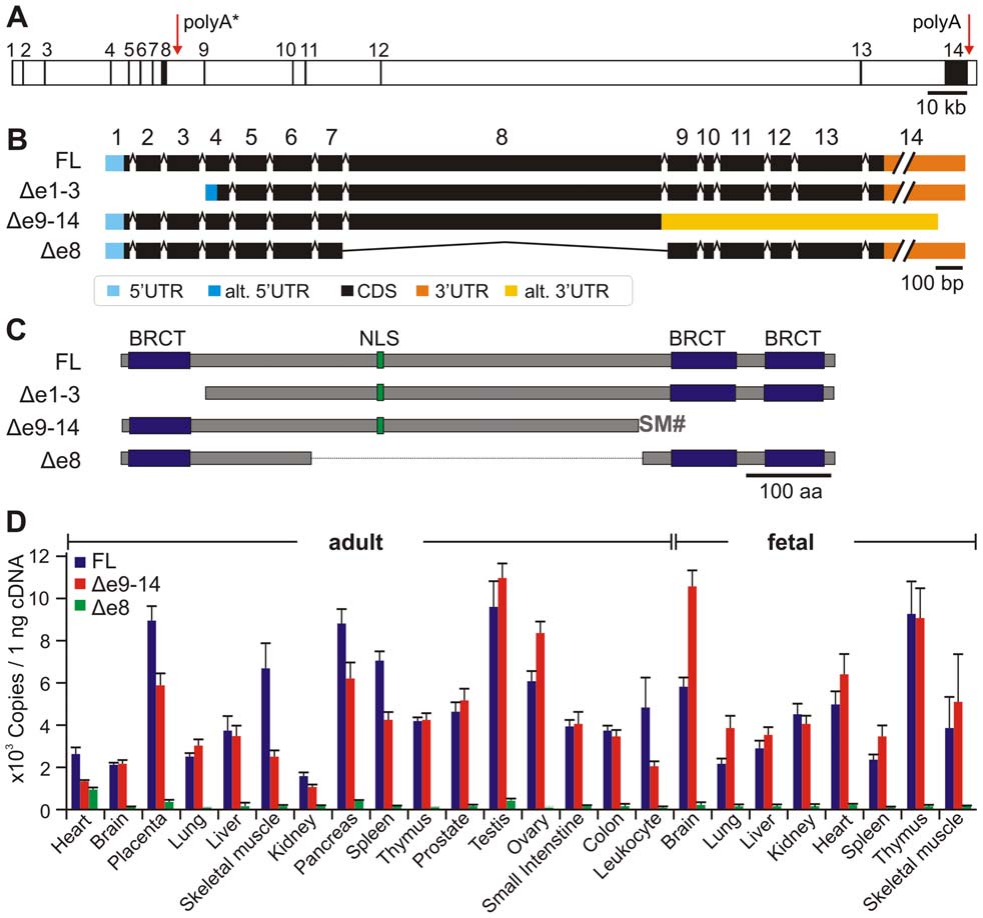
The human *MCPH1* gene, its transcripts and predicted polypeptides. (A) Exon (filled boxes) and intron (open boxes) organization of the 241 906-bp encompassing *MCPH1* gene locus. Red arrows indicate the positions of the regular and of the alternative (*) polyadenylation sites (polyA). (B) The full-length (FL) and the alternative transcripts Δe9–14, Δe1–3, and Δe8: numbered boxes indicate exons, black filled areas illustrate the entire coding regions (CDS), and colored areas show untranslated regions (UTR) as indicated. (C) Predicted polypeptides representing MCPH1 isoforms: blue boxes depict the positions of BRCT domains, while green boxes represent the site of the canonical nuclear localization signal sequence (NLS). Two additional amino acids, S and M, are included into MCPH1Δe9–14 prior to premature termination (#). (D) Expression of MCPH1 transcript variants. Columns represent the levels of MCPH1 transcripts in indicated adult and fetal tissues determined using quantitative real-time PCR. Data represent means ± one S.D. of three independent experiments and are normalized to the geometric mean levels of *UBC*, *GAPDH*, *B2M*, and *HPRT1* cDNA.

At least eight different loci (*MCPH1 – MCPH8*) are associated with MCPH [3]–[10]. Seven of the underlying genes with their products have been identified and characterized to date: *MCPH1/*microcephalin, *MCPH2/*WDR62, *MCPH3/*CDK5RAP2, *MCPH4/*CEP152, *MCPH5/*ASPM, *MCPH6/*CENP, and *MCPH7/*STIL [11]–[16]. The human *MCPH1* gene is located on chromosome 8p23.1, is organized into 14 exons and encodes a protein composed of 835 amino acids (fig. 1a).

Biallelic mutations in the *MCPH1* gene are associated with a distinctive cellular phenotype which consists of excess (10–20%) prophase-like cells (PLCs) among metaphase spreads. PLCs result from premature chromosome condensation (PCC) in the G2 phase of the cell cycle and delayed decondensation in the post-mitotic G1 phase (PCC syndrome, OMIM 60858) [17], [18]. Previous research has shown that MCPH1 acts as a negative regulator of condensin II which prevents PCC until the onset of prophase and allows timely chromosome decondensation after mitosis [19], [20]. An additional explanation for PCC involves the low levels of phosphorylated CDK1 at tyrosine residue 15 observed in *MCPH1*-mutant cells [21].

MCPH1 is predicted to contain a single N-terminal and tandem C-terminal BRCA1 carboxyl terminal (BRCT) domains in addition to a canonical nuclear localization signal (NLS) sequence [11]. BRCT domains are present in numerous proteins involved in cell cycle checkpoint functions (e.g., NFBD1/MDC1 and 53BP1) [22]. Because phosphoprotein binding domains of BRCT type are responsive to DNA damage [23], various studies have attempted to clarify the role of MCPH1 in DNA repair pathways. It has been shown that MCPH1 is recruited to lesions induced by a variety of DNA damaging agents [24]. MCPH1 is required for the formation of ionizing radiation (IR)-induced nuclear foci involving NBN, 53BP1, MDC1 and ATM and for ultraviolet light-induced foci such as ATR, RPA34, and RAD17 [25]. The C-terminal BRCT domains of MCPH1 appear to be crucial for nuclear foci formation [24]. According to studies using siRNA, downregulation of MCPH1 impairs the IR-induced intra-S-phase and G2/M checkpoints [25]. In addition, downregulation of MCPH1 expression decreases the transcript and protein levels of BRCA1 and CHK1 [26], [27]. These studies suggest that MCPH1 functions in an ATM- and ATR-dependent manner but upstream of both the ATM- and the ATR-signaling pathways. Generally, the impairment of mechanisms that regulate the function of DNA damage-induced cell cycle checkpoints and other DNA damage responses results in genomic instability and a predisposition to malignancies. In view of these findings, it is remarkable that patients whose cells have truncating mutations in *MCPH1* show normal expression levels of CHK1 and BRCA1 [21] and display a proficient G2/M checkpoint following ionizing irradiation [28]. Interestingly, none of the patients with biallelic mutations in *MCPH1* have been reported to have cancer [11], [17], [29], [30]. On the other hand, Mcph1-deficient mice have been shown to have a reduced lifespan: however, the reasons for premature death of these animals remain elusive [31]. Moreover, Liang et al. [32] observed that Mcph1 is essential for DNA repair and the maintenance of genomic stability in mice.

The multitude of functions claimed to MCPH1 could perhaps be explained by existence of different isoforms with different functions. Alternative splicing of the *MCPH1* gene has been observed in *Drosophila* [33]. In this report we investigated the expression of different transcript forms of the human *MCPH1* gene and characterized MCPH1 isoforms by comparing MCPH1-deficient cells and their stably complemented counterparts.

## Results

### Alternative transcripts of human MCPH1

We first screened the databases for the existence of alternatively spliced *MCPH1* transcripts. In addition to full-length MCPH1 (MCPH1-FL), the Alternative Splicing and Transcript Diversity database (ASTD) [34] includes a variant lacking the first three exons (MCPH1Δe1–3) and another lacking exons 9–14 (MCPH1Δe9–14) (fig. 1b). The latter is also registered as clone BC030702.1 in the GenBank database and arises from premature polyadenylation within intron 8, probably due to an alternative polyadenylation site in that intron. Indeed, a database for polyadenylation sites in vertebrate genes (polyA_DB.2) [35] includes a cluster of polyadenylation sites in intron 8 of human *MCPH1* (polyA* in fig. 1a). This premature polyadenylation results in exonization of 1 025 nt of the 5′ portion of intron 8, creating a new stop codon two codons downstream of the original exon 8 and an alternative 3′ UTR. The corresponding protein has a predicted size of approximately 70 kDa and lacks the two C-terminal BRCT domains (fig. 1c). The expression of this variant was verified by RT-PCR using a forward primer complementary to the 5′ end of *MCPH1* CDS and a reverse primer located within intron 8.

The other variant, MCPH1Δe1–3 has previously been described in a general genetic screen for negative regulators of hTERT [36]. However, this variant was first described as full-length MCPH1 and not as an alternative transcript. It would encode MCPH1 lacking the N-terminal BRCT domain. The existence of MCPH1Δe1–3 could be explained by the use of alternative transcriptional start sites (TSSs) in exon 4. To test this hypothesis, we performed 5′ RACE using RNA isolated from HeLa and 293T cells. Subcloning of the 5′ RACE products and subsequent sequencing of several clones revealed a cluster of TSSs, which were all located within exon 1. This finding is in agreement with the positions of TSSs annotated in the ASTD databank indicating that MCPH1Δe1–3 is not physiologically expressed: therefore, this variant was omitted from further experiments.

In contrast, we found a novel splicing variant of the human *MCPH1* gene using RT-PCR with primers complementary to the extreme ends of the coding region of full-length *MCPH1*. Two prominent bands with sizes of 2 500 bp and 1 300 bp were detected using agarose gel electrophoresis. Sequencing revealed that the 2 500-bp band corresponded to MCPH1-FL, while the 1 300-bp product resulted from an *MCPH1* alternative transcript that completely lacked exon 8 (MCPH1Δe8, fig. 1b). The exclusion of exon 8 causes in-frame skipping of 1 155 bp and results in a theoretical protein of 450 amino acids, with a predicted molecular weight of approximately 50 kDa. The deduced polypeptide contains all three BRCT domains but lacks the putative NLS motif, which is encoded by exon 8. The skipping of exon 8 in MCPH1Δe8 and the exonization of intron 8 in MCPH1Δe9–14 are probably due to a weak 5′ splice site in intron 8. Indeed, analyzing the 5′ splice sites of *MCPH1* introns using the splice site prediction algorithm NNSPLICE [37] revealed a score of 0.69 for the 5′ splice site of intron 8 while all the others ranged between 0.94 and 1 (table S1).

### Expression pattern of alternative human MCPH1 transcripts

Recovering of an alternative transcript is not equivalent with physiological functionality of this variant. The expression level can give a clue to the relevance of an alternative transcript. Therefore, we examined whether *MCPH1* transcripts are differently expressed in certain tissues. Quantitative real-time PCR of human fetal and adult tissues revealed that the mRNA levels of MCPH1-FL and MCPH1Δe9–14 were similarly abundant in all tissues tested (fig. 1d and table S2). Some tissues exhibited increased expression of both isoforms in the fetal stage compared with the respective adult samples (such as brain, heart, and thymus). Notably, the expression of both isoforms indicated significant differences between fetal and adult brain samples. Specifically, MCPH1Δe9–14 was overexpressed approximately five fold in the fetal brain compared with the adult brain. This observation is consistent with the role of MCPH1 in the development of the cerebral cortex. In contrast, MCPH1Δe8 was detected at low levels, comparable to background noise, indicating that this isoform is probably a byproduct of splicing events.

### Cell cycle-dependent transcription of MCPH1 isoforms

**Figure 2:**
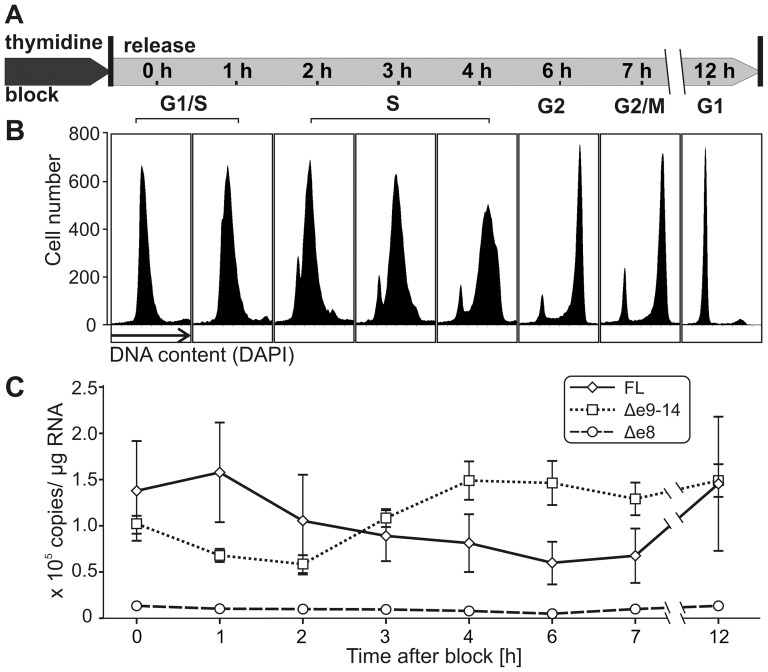
Cell cycle-dependent regulation of MCPH1 transcripts. (A) HeLa cells were arrested in G1 phase by double thymidine block. Cultures harvested at various time points after release were analyzed using flow cytometry. (B) Plots represent numbers of cells as a function of their DNA content. A total of 90% of the cells synchronously progressed into S phase (0–4 h), entered G2 phase (4–6 h), started passing through mitosis after 7 h, and were completely in G1 phase after 12 h. (C) Levels of MCPH1-FL (diamonds, solid line), MCPH1Δe9–14 (squares, dotted line), and MCPH1Δe8 (circles, dashed line) mRNA. Data represent means ± S.E.M. of three independent experiments and are normalized to the expression levels of *GAPDH* and *B2M*.

Because MCPH1 is involved in cell cycle regulation, we sought to determine if expression of *MCPH1* transcript forms was subject to cell cycle-dependent control. HeLa cells were synchronized using a double thymidine block. Cell synchrony was monitored using flow cytometry of DAPI-stained cells, and total RNA was isolated at various time points as depicted in fig. 2. Quantitative PCR demonstrated that the amount of MCPH1-FL mRNA decreased from mid-S phase to G2 phase. In contrast, the transcript levels of MCPH1Δe9–14 were downregulated during early S phase but increased during late S and G2. The combined level of MCPH1-FL and Δe9–14 transcripts did not vary significantly. Because MCPH1-FL and Δe9–14 contain the same 5′ region, the transcription of both isoforms should be regulated by the same promoter. Thus, we suppose that up- and downregulation of each variant might occur at posttranscriptional stages. MCPH1Δe8 transcripts were detectable at low levels at all-time points, supporting again the idea that this variant is a byproduct of splicing events. Transcript levels of the reference genes *GAPDH* and *B2M* were identical in all samples, indicating the validity of the observed variation in *MCPH1* expression regardless of RNA input or synchronization procedure.

### Ectopic expression of MCPH1 isoforms

**Figure 3:**
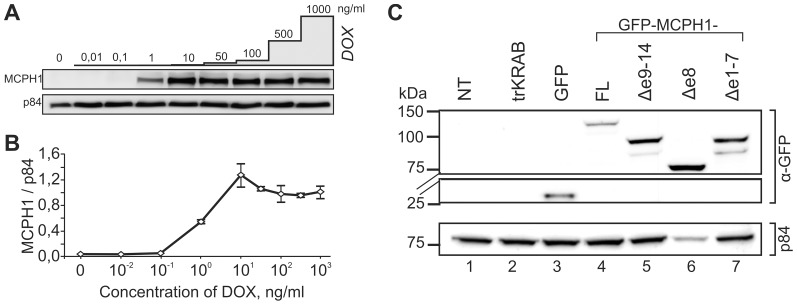
Expression of GFP-tagged MCPH1 isoforms in MCPH1-deficient 562T fibroblasts. (A) Cells were transduced with GFP-tagged coding sequence of full-length MCPH1 cDNA in a conditional, doxycycline (DOX)-dependent construct with a second regulatory construct trKRAB. Cultures were exposed to increasing DOX concentrations as indicated. Whole-cell extracts were prepared 72 h later and analyzed for the expression of MCPH1 using immunoblotting with an antibody against GFP. (B) The graph shows MCPH1 band intensity relative to the loading control p84 plotted against DOX concentrations. Data represent means ± one S.D. of three independent assays. (C) Immunoblot analysis of whole-cell extracts from non-transduced (NT) 562T cells (lane 1), 562T cells transduced with the regulatory construct only (lane 2), with GFP alone (30 kDa, lane 3) or with GFP fused to MCPH1-FL (120 kDa, lane 4), MCPH1Δe9–14 (94 kDa, lane 5), MCPH1Δe8 (78 kDa, lane 6), or MCPH1Δe1–7 (96 kDa, lane 7) with an antibody against GFP.. Nuclear matrix protein p84 served as the loading control.

Transient expression of *MCPH1* has been reported in several studies, but none of these studies used stable cell lines with ectopically expressed MCPH1. Our preliminary experiments showed that unregulated overexpression of MCPH1 causes cell death. Therefore, we expressed the three MCPH1 isoforms by cloning the respective cDNA into a lentiviral vector containing a *tet* operator (*tet*O) sequence followed by co-transduction of this vector with another vector that expressed a transcriptional repressor, the tetracycline-controlled hybrid protein tTR-KRAB [38]. Using these constructs, we obtained stable cell lines that exhibited conditional doxycycline-dependent expression of N-terminal GFP-tagged MCPH1 in transformed fibroblasts (562T) derived from a patient with the homozygous nonsense mutation *c.427dupA* (p.N143fsX147) in *MCPH1*. Western blot analysis revealed that increasing doxycycline concentrations resulted in increased GFP-MCPH1 expression (fig. 3a–b). For further experiments, we used 1 ng/ml doxycycline to induce the expression of GFP-MCPH1 fusion proteins. We checked the expression of the GFP-MCPH1 fusion proteins using immunoblotting. As shown in fig. 3c, monoclonal antibodies against GFP detected bands of the appropriate size, which were not present in samples from non-transduced cells or cells transduced only with the tTR-KRAB vector. Similar results were obtained using commercially available antibodies against MCPH1. While the commercial antibody AF3998 detected all three isoforms, another such antibody, ab2612, failed to detect MCPH1Δe8 because it lacks the antigenic epitope located within the inter-BRCT region (data not shown).

### Complementation of the PCC phenotype in MCPH1-deficient cells

**Figure 4:**
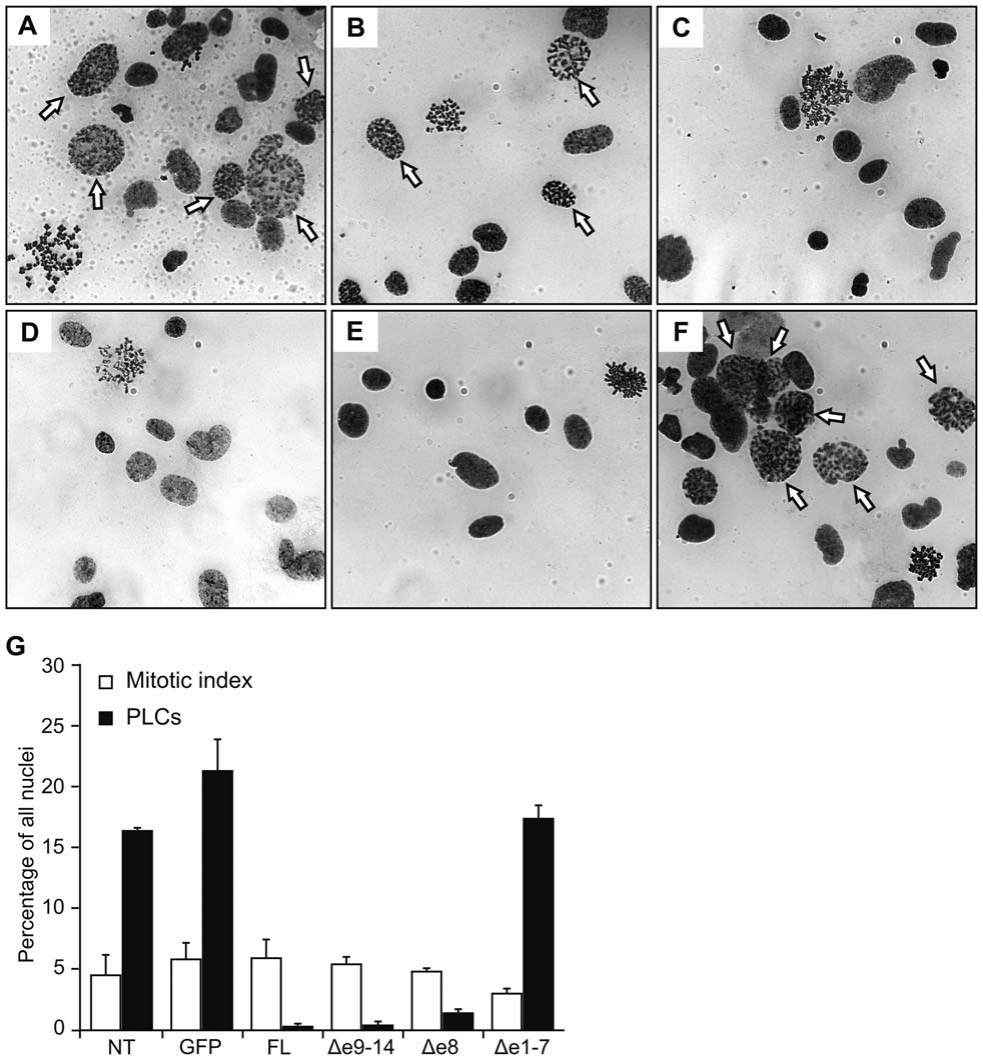
Complementation of PCC in patient fibroblasts. Cells are derived from patient with homozygous truncating mutation c.427dupA (p.T143NfsX5) in *MCPH1*. Chromosome preparations from (A) non-transduced cells and (B) cells expressing GFP only, or GFP fusions with (C) full-length, (D) Δe9–14, (E) Δe8, or (F) Δe1–7 MCPH1. Arrows indicate nuclei of prophase-like cells (PLCs). (G) Mean rates of PLCs (filled columns) of slides from A-F. Open columns represent mean mitotic indices. Error bars denote the S.D. of counts of approximately 1000 cells each from three independent experiments.

To analyze the ability of MCPH1 isoforms to complement the defective chromosome condensation in MCPH1-deficient 562T cells, chromosome spreads were prepared from 562T cells expressing the GFP-MCPH1 isoforms, and the proportion of prophase-like cells (PLCs) was determined (fig. 4). Interestingly, all three isoforms were able to complement the PCC phenotype (fig. 4c–e). While the proportion of PLCs in non-transduced cells (16%) or in cells expressing GFP alone (21%) was typical for PCC syndrome, cells expressing MCPH1-FL, Δe9–14, or Δe8 exhibited a reduced number of PLCs (0.3%–1.3%) in the normal range. Mitotic indices were similar in all samples (5%±0.8%, fig. 4g), confirming that the reduction in the proportion of PLCs was not due to reduced proliferative activity.

Comparing the protein sequences of the various MCPH1 isoforms revealed that the N-terminal BRCT domain was common to all three variants (fig. 1c). For this reason and considering that all of the mutations described in MCPH1 patients are located within the first seven exons, we created a deletion construct lacking exons 1–7 (MCPH1Δe1–7) and subcloned it into the same vector described above. This particular construct, predicted to encode an MCPH1 isoform with a deleted N-terminal BRCT domain failed to complement the PCC phenotype, showing the same proportion of PLCs (17%, fig. 4f) as non-transduced 562T cells, confirming the importance of this region of MCPH1 for the proper condensation of chromosomes.

### Only simultaneous downregulation of MCPH1-FL and Δe9–14 cause the cellular phenotype of PCC

**Figure 5:**
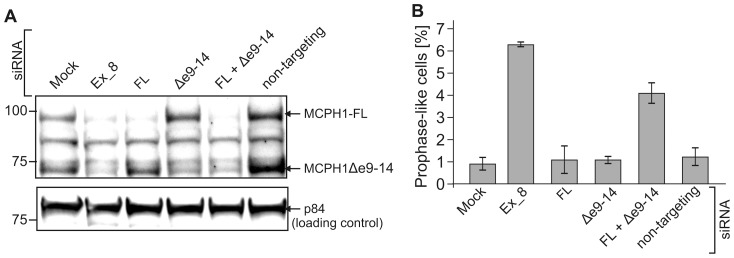
Only simultaneous downregulation of endogenous full-length (FL) and Δe9–14 MCPH1 induces PCC. (A) Immunoblots with an anti-MCPH1 antibody after transfection of HeLa cells with different siRNAs demonstrate efficient downregulation of either MCPH1-FL (93 kDa) or MCPH1Δe9–14 (70 kDa) or both isoforms (exon 8 and FL+ Δe9–14) but no downregulation in response to non-targeting siRNA or mock-transfected controls. Nuclear matrix protein p84 served as a loading control. (B) Columns represent PLC rates in HeLa cells transfected with MCPH1 siRNA as indicated in A. Error bars denote the S.D.

Trimborn *et al*. [18] have previously shown that the downregulation of MCPH1 using RNAi causes the cellular phenotype of misregulated chromosome condensation in HeLa cells. The siRNA sequence used by Trimborn *et al.* was directed against exon 8 of the *MCPH1* gene, making it able to deplete both FL and Δe9–14 transcripts. In an extension of these previous studies, we investigated whether the downregulation of each variant individually would affect chromosome condensation. Transcript Δe8 was omitted from this experiment because of its marginal expression levels. Because MCPH1-FL and Δe9–14 contain different 3′ UTR sequences (fig. 1b), siRNA specific to these sequences allowed us to downregulate each variant individually. HeLa cells were transfected with siRNA duplexes specific for exon 8 of *MCPH1* to deplete the both transcripts, or to the 3′ UTRs of FL or Δe9–14 to deplete them separately. Downregulation of MCPH1 was monitored using immunoblotting with an MCPH1-specific antibody (fig. 5a). Discrete bands at approximately 100 kDa and 75 kDa were detected, which correspond to MCPH1-FL and Δe9–14, and disappeared when the cells were treated with siRNA specific to the 3′ UTR of FL or Δe9–14, respectively. As expected, the siRNA against exon 8 of *MCPH1* or the combination of isoform specific siRNAs led to the downregulation of both variants.

As shown in fig. 5b, individual downregulation of each of the variants failed to affect the regulation of chromosome condensation. Only the combined depletion of both variants induced the PCC cellular phenotype. This observation is fully consistent with the results of the complementation assays described above, which show that both isoforms are redundant regarding the regulation of chromosome condensation.

### Intracellular localization of MCPH1 isoforms

Centrosomal localization has been described for human MCPH1 in U2OS cells and for chicken cMCPH1 in DT40 cells [39], [40]. We examined the centrosomal localization of MCPH1 isoforms in 562T and HeLa cells, both expressing GFP-MCPH1, using antibodies against the centrosomal proteins γ-tubulin and pericentrin. Surprisingly, there was no evidence for the centrosomal localization of any of the ectopically expressed MCPH1 isoforms (fig. S1 and S2).

**Figure 6:**
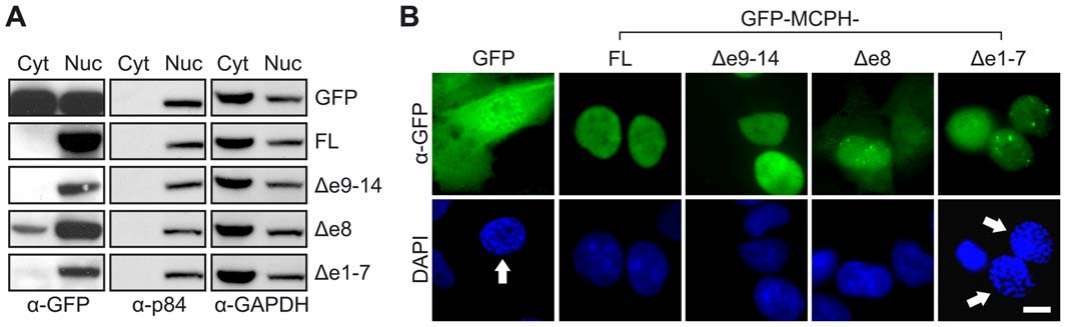
Intracellular distribution of MCPH1 isoforms. (A) MCPH1-deficient fibroblasts expressing GFP alone or the specified GFP-MCPH1 fusion proteins were fractionated and cytoplasmic (Cyt) and nuclear (Nuc) protein extracts were analyzed using immunoblotting with an antibody against GFP. The nuclear matrix protein p84 and GAPDH were used as index proteins and loading controls. (B) Cells indicated in A stained with an anti-GFP antibody (green), counterstained with DAPI (blue) and analyzed using fluorescence microscopy. Arrows indicate the prophase-like nuclei. Scale bar = 10 µm. All MCPH1 isoforms exhibit unambiguous nuclear localization.

To gain insight into the subcellular localization of the MCPH1 isoforms, 562T fibroblasts transduced with GFP fusion constructs were fractionated, and proteins extracted from the nuclear and cytoplasmic fractions were analyzed using immunoblotting (fig. 6a). The purity of each fraction was confirmed using antibodies against the nuclear matrix protein p84 and GAPDH, respectively. Expression of GFP alone demonstrated the expected distribution in both the cytoplasm and the nucleus. In contrast, all GFP-tagged MCPH1 variants were predominantly located in the nucleus-derived cell fraction. Although low levels of MCPH1Δe8 were detected in the cytoplasm, a more intense signal even from this isoform was seen in the nucleus despite the predicted absence of the canonical NLS. Importantly, the mutant variant MCPH1Δe1–7 could be localized in nucleus as well demonstrating that the failure of this particular variant to complement the PCC phenotype is not due to defective nuclear targeting. As shown in fig. 6b, these results were confirmed using fluorescence microscopy of DAPI-stained cells.

### NLS analysis of MCPH1

**Figure 7:**
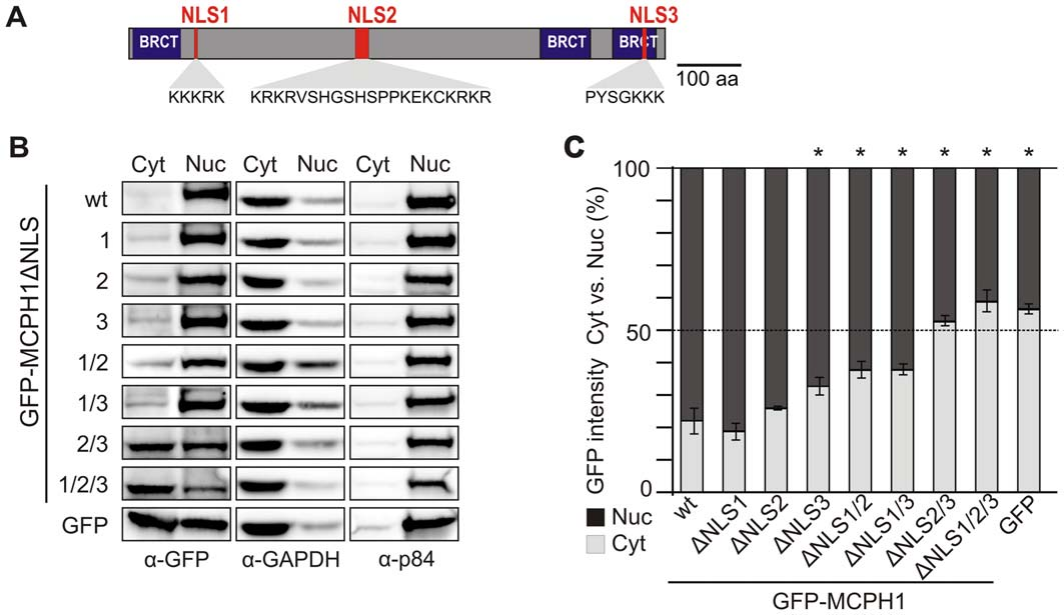
Nuclear localization signals (NLSs) in human MCPH1. (A) The positions of the putative NLS motifs and their amino acid sequences are highlighted in the diagram of the full-length MCPH1. (B) Subcellular distribution of GFP-tagged wild-type (wt) MCPH1 and mutants with deleted NLSs as indicated were transiently expressed in HeLa cells. Cytoplasmic (Cyt) and nuclear (Nuc) protein extracts were immunoblotted with an antibody against GFP (left panel). GAPDH (center panel) and the nuclear matrix protein p84 (right panel) served as index proteins and loading controls. (C) Ratios of relative GFP band intensity in the cytoplasmic (Cyt) vs. nuclear (Nuc) fractions. Absolute numbers were assessed using densitometry and normalized to the loading controls. Columns designate means, and error bars represent the S.D. from three different experiments. Significant differences to wt MCPH1 are indicated by asterisks denoting *p*<0.05 (Student's *t*-test). Scale bar = 10 µm.

To explain the nuclear targeting of MCPH1Δe8, we employed an *in silico* screen of the MCPH1 protein for NLS motifs using the PSORT II prediction program [41]. In addition to the canonical bipartite NLS encoded by the exon 8 motif KRKRVSHGSHSPPKEKCKRKR, we identified two further motifs with predicted NLS functions (KKKRK, localized at the N-terminus, and PYSGKKK, localized at the C-terminus; fig. 7a). We designated these three putative nuclear localization signals NLS1, NLS2 and NLS3. Using PCR-based *in vitro* mutagenesis, variants with different combinations of deleted NLS motifs in GFP-MCPH1 fusion constructs were generated for transfection into HeLa cells. GFP expressed from a control pEGFP-N3 vector was distributed evenly between the cytoplasm and the nucleus, whereas GFP fused to wild-type (wt) MCPH1 was detected almost exclusively (78%±4%) within the nuclear fraction (fig. 7b–c). Deletion of NLS1 or NLS2 alone had no effect on nuclear localization, whereas MCPH1 with a deleted NLS3 shifted slightly but statistically significant (*p*<0.05, *t*-test versus wt MCPH1) the GFP distribution to the cytoplasmic fraction. Pairwise deletions of NLS1 with NLS2 or NLS3 did not change noticeably the localization of mutated isoforms, however, in comparison to wt MCPH1 the effect was significant (*p*<0.05, *t*-test). Finally, the absence of NLS2 and NLS3 or of all three NLS motifs consistently affected the nuclear localization of MCPH1 (44% of the GFP signal within the nucleus; *p*<0.05 versus wt MCPH1). The purity of each fraction was controlled using antibodies against the nuclear matrix protein p84 and GAPDH. Similar results were obtained using transiently transfected COS-7 and 293T cells (data not shown).

### Formation of repair foci by MCPH1 isoforms

Using fluorescence microscopy of 562T fibroblasts ectopically expressing the GFP-fused MCPH1 isoforms, we observed MCPH1-FL and MCPH1Δe8 in punctuate nuclear speckles in 10.8% and 4.8% of cells, respectively, whereas MCPH1Δe9–14 was evenly distributed in the nucleus. In addition, the mutant isoform Δe1–7 also formed foci (in 5.8% of cells), indicating the requirement of C-terminal BRCT-domains for foci formation (data not shown).

**Figure 8:**
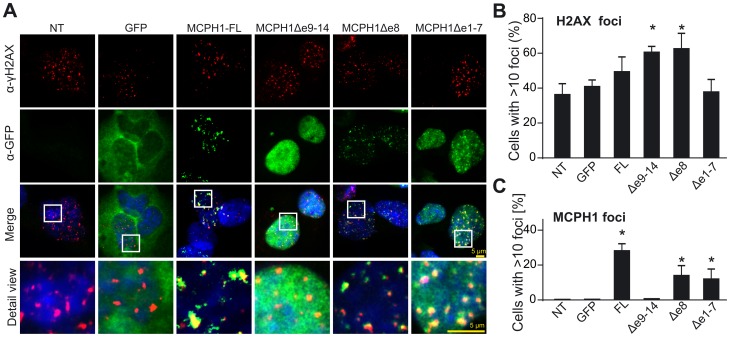
Colocalization of MCPH1 and γH2AX in ionizing irradiation-induced nuclear foci. (A) Non-transduced (NT) MCPH1-deficient 562T cells and 562T stably expressing GFP alone or the specified GFP-MCPH1 fusion proteins were fixed 2 h after irradiation with 10 Gy and co-stained with antibodies against γH2AX (red) and GFP (green). Nuclei were counterstained with DAPI (blue). Rectangles frame areas, which are shown enlarged in the bottom row. MCPH1 focus formation was observed for MCPH1 isoforms containing the C-terminal BRCT tandem. (B) Quantification of cells expressing foci containing γH2AX and/or (C) MCPH1. Error bars indicate the S.D. of three different measurements, counting approximately 300 nuclei. * *p*≤0.05 vs. NT as calculated using the Student's *t*-test.

Because it is known that MCPH1 relocates to sites of DNA damage [24], [27], we examined which of the MCPH1 isoforms was involved in IR-induced foci formation. In this experiment, we irradiated 562T cells ectopically expressing the GFP-fused MCPH1 isoforms with 10 Gy and analyzed the formation of MCPH1 and γH2AX foci two hours later. As expected, MCPH1 foci were formed by MCPH1-FL, Δe8 or Δe1–7 but not the isoform Δe9–14, which lacks the C-terminal BRCT tandem (fig. 8a). Interestingly, in cell lines expressing Δe8 or Δe1–7, fewer cells (13.7% and 11.7%) showed MCPH1 foci formation compared with lines expressing MCPH1-FL (28%).

In contrast, γH2AX foci were detected in all samples, indicating that the formation of γH2AX foci is independent of MCPH1 expression or MCPH1 foci formation (fig. 8b–c). Interestingly, however, in cell lines ectopically expressing MCPH1-FL, Δe9–14 or Δe8, we observed elevated γH2AX foci formation (49%, 60% and 62% of the total cell population, respectively) when compared with cells expressing only GFP or the Δe1–7 variant (40.6% and 37.5% of the total cell population, respectively).

## Discussion

The *MCPH1* gene is mutated in patients with primary microcephaly and misregulated chromosome condensation. The existence of BRCT domains in MCPH1 and studies using RNAi against MCPH1 suggest a role for MCPH1 in cell-cycle checkpoint control and in the DNA damage response [21], [24], [42]. These previous studies assumed that there was a single MCPH1 isoform. However, the multitude of functions attributed to MCPH1 suggests the existence of multiple MCPH1 isoforms. Alternative splicing of pre-mRNA allows the formation of multiple transcripts from a single gene, contributes to proteome diversity, and provides a level of gene regulation [43]. Of note, 46% of alternatively spliced genes are involved in signal transduction or gene expression regulation [44]. In addition, alternative transcription initiation and alternative choices of polyadenylation sites further enhance proteome diversity [45].

Here we provide evidence for two additional isoforms of MCPH1 produced by alternative splicing and alternative polyadenylation. One of these novel isoforms, MCPH1Δe9–14, was found to be as abundant as the full-length transcript in several adult and fetal tissues, suggesting the functionality of this particular isoform. In contrast, the low copy number detected for MCPH1Δe8 argues against relevance of this variant. However, we found that both novel isoforms were able to complement the defective chromosome condensation in cells derived from MCPH1 patients. Apparently, the N-terminal BRCT domain of MCPH1 is sufficient to rescue the PCC phenotype in MCPH1-deficient cells. This finding is consistent with results obtained from studies of MCPH1^−/−^ mouse embryonic fibroblasts (MEFs), which also recapitulate the defective chromosome condensation observed in human MCPH1-deficient cells [46]. Similar to our results, the PCC defect in MCPH1^−/−^ MEFs could be rescued by wt MCPH1 or variants containing the N-terminal BRCT domain. In a recent study the X-ray crystal structure of this domain was elucidated [47]. The N-terminal BRCT domain of MCPH1 contains a hydrophobic pocket in the equivalent structural position to the phosphate binding site found in other BRCT domains. Mutations in this pocket abrogate the ability of full length Mcph1 to rescue the PCC phenotype in MCPH1^−/−^ MEFs. Moreover, Yamashita et al. [20] investigated the specific role of hMCPH1 in regulation of chromosome condensation using *Xenopus* egg extracts. They found that hMCPH1 inhibits loading of condensing II by competing for its binding sites on chromosomes and that the N-terminal domain of hMCPH1 is required and sufficient for this inhibitory activity. These data additionally support our findings on the importance of the N-terminal BRCT domain for the timely condensation and decondensation of chromosomes.

In contrast to previous reports with U2OS cells [39], [48], we were unable to confirm a centrosomal localization for any of our GFP- or FLAG-tagged MCPH1 isoforms in HeLa cells or SV40-transformed 562T fibroblasts. This discrepancy may be due to tissue or cell-type specificity. In addition to cell line differences, there is the possibility of uncharacterized isoforms, which could interact with centrosomes. However, it is important to note that the *Drosophila melanogaster* homolog of *MCPH1* expresses two alternatively spliced isoforms mcph1(L) and mcph1(S) [49], which correspond to human MCPH1-FL and MCPH1Δe9–14. A centrosomal localization for mcph1(S) was suggested but could not be confirmed in the *Drosophila* studies [33], [49]. In contrast, nuclear localization was observed for both *Drosophila* variants, which is fully consistent with our findings on the subcellular distribution of the human MCPH1 isoforms. While we were preparing our manuscript, the causative gene located in the *MCPH2* locus was identified. Interestingly, similar contradictory results are reported concerning the localization of *MCPH2* product WDR62: while two studies detected WDR62 localization at mitotic spindles [12], [50] another group could not confirm the centrosomal localization of this protein [51].

The unambiguous nuclear localization of all tested human MCPH1 isoforms is fully in line with the role of MCPH1 in DNA damage response and with the presence of multiple NLS motifs sufficient for targeting these isoforms to the nucleus. Multiple karyophilic signals with independent activity are common among nuclear proteins [52], [53], maintaining nuclear transport in the event of point mutations in one of the NLSs. In addition, a multitude of NLSs may increase the rate of protein-transporter interaction, whereby transport through the nuclear pores occurs more effectively [54]. The location of NLS3 within the third BRCT domain of MCPH1 is consistent with other features common to nuclear proteins because NLS motifs are frequently localized in close proximity to DNA-binding or protein-interaction domains [55]. While the N-terminal domain of MCPH1 seems to be indispensable for the rescue of defective chromosome condensation in MCPH1-deficient cells the C-terminal BRCT tandem appears essential for the localization of MCPH1 to sites of DNA damage. Our data confirm previous findings which demonstrated that the ability of MCPH1 to localize to the sites of DNA double-strand breaks (DSBs) depends on its C-terminal tandem BRCT domain [24], [40]. Using ITC assay, Wood et al. [24] also reported that MCPH1 tandem BRCT domains bind tightly to a phospho-H2AX peptide *in vitro*. However, our data also suggest that the inter-BRCT region and the N-terminal BRCT domain ensure further the MCPH1 foci formation in response to IR-induced DNA lesions. It is conceivable that MCPH1-FL is targeted (via its C-terminal BRCT tandem) to the sites of DNA damage, while Δe9–14 is responsible for proper condensation and decondensation of chromosomes during the cell cycle. This suggestion would mean that the two MCPH1 isoforms have distinctive functions. However, only simultaneous downregulation of both isoforms consistently induced the PCC cellular phenotype. Obviously, the two isoforms can complement each other in some functions (e.g., chromosomal condensation) but not in others (e.g., response to DNA damage).

One paradigmatic case resembling MCPH1 is the human promyelocytic leukemia protein (PML), which exists in multiple isoforms generated by alternative splicing that differ in their C-terminal regions. While isoform PML-III interacts with the centrosome, the others are localized in the nucleus and serve different functions [56]. Similar to MCPH1, PML is involved in multiple cellular activities, such as the DNA damage response, apoptosis and chromatin modification. Furthermore, a recent study showed that loss of Pml in mice resulted in decreased cortical thickness and brain size [57]. Therefore, it remains to be seen whether additional isoforms of MCPH1 with additional functions may exist in human cells.

The alternative transcript MCPH1Δe9–14 exhibits a different 3′ UTR, enabling a putative differential regulation of expression by means of microRNAs (miRNAs). This notion is supported by the finding that expression of MCPH1-FL and Δe9–14 alternate in a cell cycle-dependent manner and independently of each other. An algorithm for the detection of miRNA binding sites (MicroInspector) revealed binding sites for 97 different miRNAs in the 3′ UTR of MCPH1-FL and 52 in the Δe9–14 transcript, with only 11 miRNAs common to both variants. It has been shown that the binding of miRNAs to the 3′ UTRs of target genes stimulates rapid degradation of the target transcript [58]. Therefore, future studies are required to investigate if different miRNA-mediated specificities may also apply for the regulation of MCPH1 isoforms.

In summary, our results provide new insights into expression pattern of human *MCPH1* gene contributing to better understanding of different functions attributed to the gene product.

## Materials and Methods

### Cell culture

Adherent cell lines were grown in a monolayer in minimum essential medium (MEM) (Invitrogen, Carlsbad, CA, USA) supplemented with 10% fetal bovine serum (FBS) and maintained at 37°C in 5% CO_2_. The 562T cell line is derived from patient fibroblasts and contains the homozygous nonsense mutation *c.427dupA* (p.N143fsX147) in *MCPH1* [18]. HeLa cells were obtained from DSMZ (Braunschweig, DE). COS-7 and 293T cells were obtained from ATCC (Manassas, VA, USA).

### Cell synchronization

A double thymidine block was used for HeLa cell synchronization. Cells were first incubated with 2 mM thymidine for 18 h. After the first thymidine block, cells were incubated in normal culture medium for 9 h, and thymidine (2 mM) was then added for a further 17 h. This procedure synchronized cells at the G1/S border. Cell cycle arrest was subsequently released by growing cells in thymidine-free medium. After release, cells were harvested in 1-h intervals for 12 h. The synchrony of HeLa cells was monitored using flow cytometry of DAPI-stained cells.

### Molecular cloning of MCPH1 coding sequence

Full-length and alternatively spliced sequences of human *MCPH1* were isolated from HeLa cells with specific PCR primers and Phusion polymerase (Finnzyme, Espoo, FI). PCR products were cloned in-frame with the EGFP cDNA being a 5′tag for the MCPH1 into lentiviral vector pCL1THPC.EGIN, thereby creating the vector pCL1THPC.EGMCPH1IN. The neomycin resistance gene (*ncpt II*) was co-expressed with the fusion protein via an EMCV IRES sequence. The vector also contained the *tetO* sequence in the 3′LTR which upon proviral integration of the vector into the target cell genome was copied also to the 5′LTR and therefore allowed better regulation of the protein expression via doxycyclin. Constructs were verified by sequencing.. The sequence of human *MCPH1* is available in the Ensembl data base under accession number ENSG00000147316. Detailed cloning strategies, vector maps and primer sequences are available upon request.

### Lentiviral transduction and complementation of MCPH1-deficient cells

HEK 293T cells were transfected using FuGene HD (Roche) with 6 µg of an HIV1 helper plasmid pCD/NL-BH, kindly obtained from Jakob Reiser, New Orleans, expression construct for HIV1 gag/pol/rev (pCD/NL-BH) [59], 6 µg of the envelope vector (pczVSV-G) [60], and 6 µg of the vector plasmids pCL1THPC.EGMCPH1IN (doxycycline-dependent transgene with EGFP tag and IRES-NeoR cassette) or puc2CL1tTRKRABIH (KRAB-based tet repressor with IRES-HygroR cassette), respectively. Viral supernatants were harvested 48 h after transfection, filtered through a 0.45-µm filter (Sartorius AG, Germany) and used to co-transduce 562T fibroblasts and HeLa cells. The selection of cells with integrated copies of the tTR-KRAB-expressing vector was accomplished using hygromycin B (200 µg/ml) selection at 24–48 h following transduction. After successful selection, doxycycline (1 ng/ml) was added to induce the expression of GFP or GFP-MCPH1 and the Neo resistance gene. Selection with G418 (350–400 µg/ml) was started 48 h after incubation with doxycycline and continued until the nontransduced cells had died. Complementation of defective chromosome condensation in transduced MCPH1-deficient fibroblasts was verified using chromosome analysis. Chromosome preparation was performed using standard methods, and >1000 nuclei per preparation were scored. The percentage of prophase-like cells was determined counting nuclei with condensed chromosomes and an intact nucleus. Mitotic index was determined counting the metaphase spreads as described previously [14].

### Antibodies

Rabbit antiserum (ab2612) and goat antiserum (AF3998) to MCPH1 were obtained from Abcam (Cambridge, UK) and R&D Systems (Minneapolis, MN, USA), respectively. The rabbit polyclonal antiserum against MCPH1 (fig. 5) was kindly furnished by Dr. Tatsuya Hirano (RIKEN, JP). Antibodies against GFP (ab13970), pericentrin (ab4448), γ-tubulin (ab11316), γ-H2AX (pS139, ab11174), nuclear matrix protein p84 (ab487), and GAPDH (ab9385) were obtained from Abcam. Mouse anti-histone H3 (pS10, #9706) was obtained from Cell Signaling (Danvers, MA, USA); A rabbit anti-γ-tubulin antibody (T5192) was obtained from Sigma (St. Louis, MO, USA). Alexa Fluor-labeled secondary antibodies were obtained from Invitrogen. HRP-conjugated secondary antibodies were from GE Healthcare (Little Chalfont, UK).

### Site-specific mutagenesis by overlap extension

To delete several NLSs in the wild-type (wt) MCPH1 we performed mutagenesis by overlap extension, as previously described [61]. The cloned coding region of full-length *MCPH1* in the S11IN vector was used as the template. In the first step, two fragments of the gene sequence were amplified in two separate PCRs. Each reaction used one flanking primer that hybridizes at one end of the target sequence and one internal primer that hybridizes to the site of the mutation with the desired deletion. Following PCR, the wt sequence was removed by cleavage with *Dpn*I. Because the two internal primers used in the first PCR overlapped, the two generated fragments could be fused in a subsequent primer-extension reaction. The obtained sequences were digested with *Bsr*GI and subcloned into the mammalian expression vector pEGFP-N3 (Clontech, Mountain View, CA, USA), which allows for in-frame fusion with enhanced GFP expression. The orientation and correctness of the constructs were verified by sequencing. Primer sequences are available on request.

### Cell transfection and subcellular localization

For subcellular detection of the GFP-tagged proteins, HeLa or COS-7 cells were cultured in 6-well plates in 3 ml MEM medium containing 10% FBS and transfected with 1 µg of the respective plasmid using 3 µl GeneJuice® Transfection Reagent (Novagen/Merck, Darmstadt, DE) according to the manufacturer's instructions. After 48 h, cells were harvested and analysed using cell fractionation or immunofluorescence.

### RNA interference

siRNA-mediated depletion of MCPH1 was performed as previously described [18].

### Whole-cell extracts and cell fractionation

For the preparation of whole-cell extracts, cell pellets were washed in cold PBS and resuspended in lysis buffer (50 mM Tris, 150 mM NaCl, 2 mM EDTA, 2 mM EGTA, 25 mM NaF, 0.1 mM NaVO_2_, 25 mM ß-glycerophosphate, 0.2% (v/v) Triton® X-100, 0.3% (w/v) Nonidet P-40, and protease inhibitor cocktail (Roche Diagnostics, Mannheim, DE)). Cells were lysed on ice for 45 min. The separation of nuclear extracts from cytoplasmic fractions was performed using a Nuclear/Cytosol Extraction Kit (BioVision, Mountain View, CA, USA). Aliquots of equal amounts of protein were mixed with NuPAGE® sample buffer (Invitrogen), heated at 70°C for 10 min, and electrophoresed for western transfer using the NuPAGE® system (Invitrogen), according to the manufacturer's instructions. Membranes were blocked, incubated with the indicated antibodies, and developed using enhanced chemiluminescence.

### Immunofluorescence

For immunofluorescence, cells grown on glass coverslips were fixed either in −20°C methanol/acetone (1∶1) for 7 min to preserve centrosomes or, in other experiments, with 4% paraformaldehyde in PBS (pH 7.4) for 15 min at room temperature and permeabilized with ice-cold methanol for 30 min on ice. Cells were incubated with PBS containing 20% FBS as a blocking agent for 30 min and then with the indicated antibodies for approximately 1 h at room temperature. After being washed three times with PBS, cells were incubated with the respective secondary antibodies conjugated with Alexa Fluor 488 or Alexa Fluor 594 for 30 min. DNA was stained with 1 µg/ml DAPI for 5 min. Following a PBS rinse, coverslips were mounted with ProLong antifade reagent (Invitrogen). Fluorescence images were captured and processed using the inverse microscope Axiovert 200 M equipped with the Plan Apo 63×/1.4 oil immersion objective and AxioVision software (Carl Zeiss, Jena, DE).

### Real-time PCR

Total RNA was isolated using an RNeasy mini kit (Qiagen, Hilden, DE). DNA contamination was removed by treating the RNA samples with the Turbo DNA-free™ Kit (Ambion, Austin, TX, USA). First-strand synthesis was performed using a Transcriptor First Strand cDNA Synthesis Kit (Roche). A total of 1 µl of each first-strand sample was used for real-time PCR, which was performed using PerfeCTa™ SYBR® Green SuperMix (Quanta BioSciences, Gaithersburg, MD, USA) and the iQ Real-Time PCR System (Bio-Rad, Hercules, CA, USA) according to the manufacturer's recommendations. A total of 48 cycles were performed (each cycle consisted of 15 s at 95°C, 30 s at 60°C and 30 s at 72°C). *UBC-*, *B2M-*, *HPRT-*, and *GAPDH-*specific primers (PrimerDesign, Southampton, UK) were used as endogenous references. All samples were analyzed in duplicate. A dilution series of each *MCPH1* variant cloned in a particular vector was used for the creation of copy number standard curves. Human Multiple Tissue cDNA panels I, II and fetal (Clontech) were used for the comparison of the expression of *MCPH1* isoforms in different tissues. Primer sequences were as follows: FL-fw 5′-ttatagttgactgtaacatggagacg-3′; FL-rev 5′-atgaggtttgatgaggtccttaa-3′; Δe8-fw 5′-gtgatactttgtgttcaggtgtt-3′; Δe8-rev: 5′-tttgcctctcccacttttctt-3′; Δe9–14-fw 5′-acttgctgtcttgtggaacttcta-3′ and Δe9–14-rev: 5′-acttcactcccctcagttattcatact-3′.

### 5′ RACE assay

5′ RACE was performed using a FirstChoice® RLM-RACE kit (Ambion) according to the manufacturer's instructions.

## Supporting Information

Figure S1 **MCPH1 do not colocalize with centrosomes.** Non-transduced (NT) fibroblasts or fibroblasts with ectopic expression of GFP or of GFP fusions with full length (FL) MCPH1 or with the indicated isoforms were methanol fixed and stained with γ-tubulin-specific antibody (red) to visualize the centrosomes. MCPH1 isoforms were detected via its GFP tag (green). Squares frame areas for a detailed view displayed in the upper right corner. Please note the clear nuclear localization of MCPH isoforms. Scale bar = 5 µm.
(TIF)

Figure S2 **FLAG-tagged MCPH1 do not colocalize with centrosomes.** Non-transduced (NT) HeLa cells or HeLa cells transiently expressing FLAG or FLAG-tagged full length (FL) MCPH1 or the indicated isoforms were methanol fixed and stained with a γ-tubulin-specific antibody (red) to visualize centrosomes. MCPH1 isoforms were detected via its FLAG-tag (green), nuclei were visualized by DAPI staining (blue). Merging the signals depicts the missing colocalization of MCPH1 with centrosomes (lower panel), a detailed view of the centrosomes is shown in the upper right corner. Scale bar = 10 µm.
(TIF)

Table S1 **Computed scores representing predicted strengths of function of the canonical splice donors in human** ***MCPH1.***
(DOCX)

Table S2 **Expression of MCPH1 splicing variants in human adult and fetal tissues.**
(DOCX)
