# Supplementary figures and images for: A Novel MCPH1 Isoform Complements the Defective Chromosome Condensation of Human MCPH1-Deficient Cells
Source: PLoS One. 2012 Aug 30;7(8):e40387. doi: 10.1371/journal.pone.0040387 (PMC3431399; doi:10.1371/journal.pone.0040387)

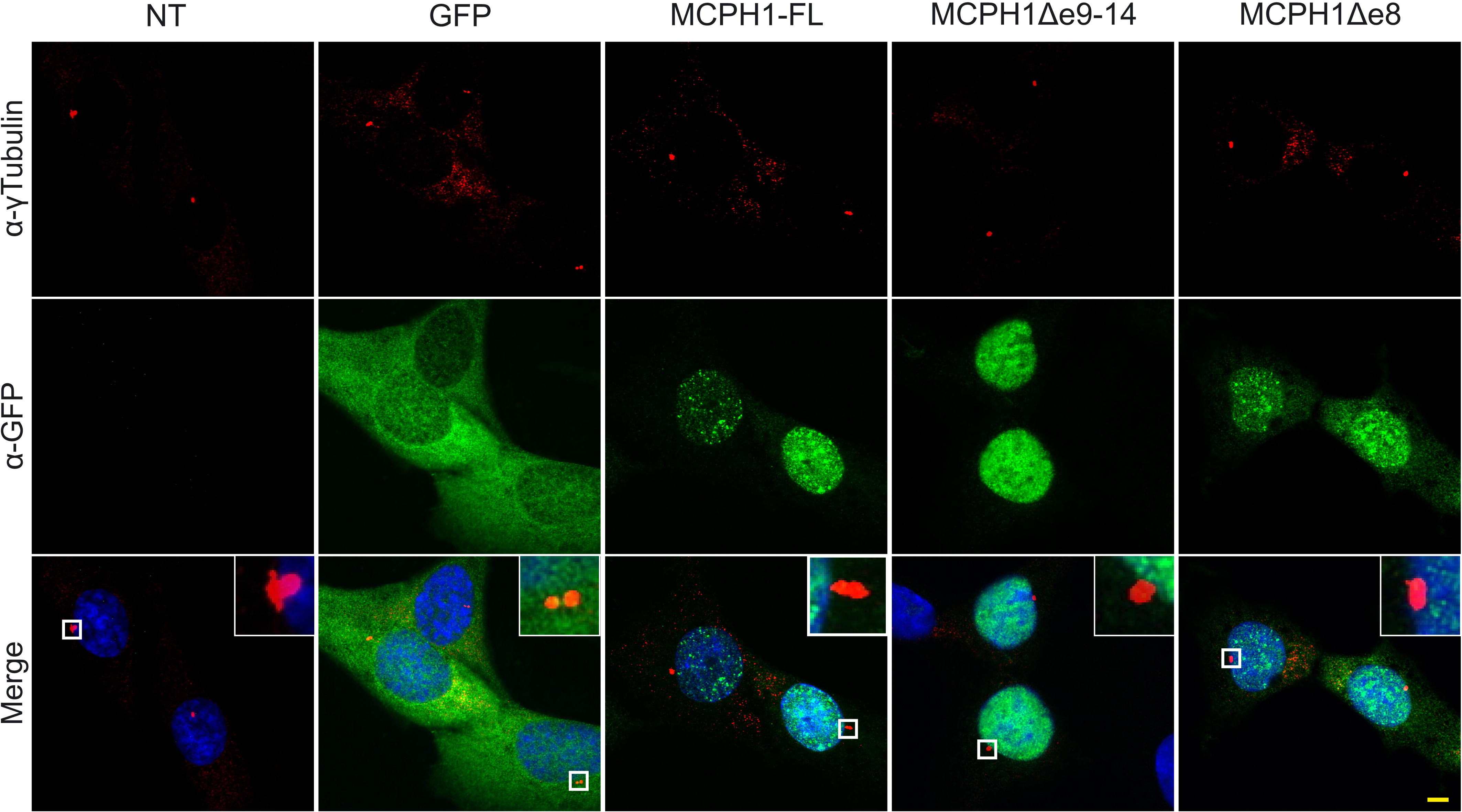

Supplement: Figure S1 — MCPH1 do not colocalize with centrosomes. Non-transduced (NT) fibroblasts or fibroblasts with ectopic expression of GFP or of GFP fusions with full length (FL) MCPH1 or with the indicated isoforms were methanol fixed and stained with γ-tubulin-specific antibody (red) to visualize the centrosomes. MCPH1 isoforms were detected via its GFP tag (green). Squares frame areas for a detailed view displayed in the upper right corner. Please note the clear nuclear localization of MCPH isoforms. Scale bar = 5 µm. (TIF) [file pone.0040387.s001.tif]

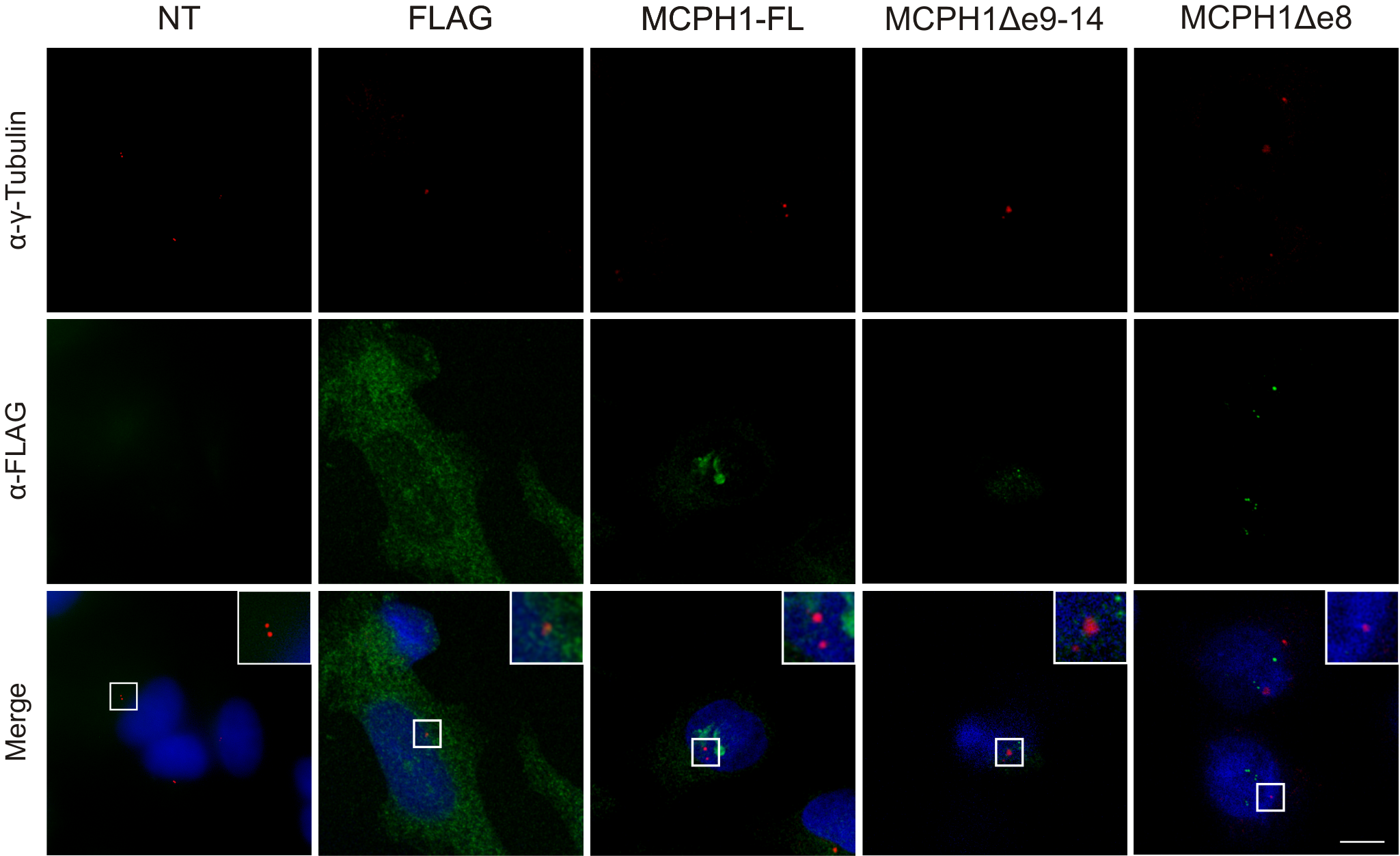

Supplement: Figure S2 — FLAG-tagged MCPH1 do not colocalize with centrosomes. Non-transduced (NT) HeLa cells or HeLa cells transiently expressing FLAG or FLAG-tagged full length (FL) MCPH1 or the indicated isoforms were methanol fixed and stained with a γ-tubulin-specific antibody (red) to visualize centrosomes. MCPH1 isoforms were detected via its FLAG-tag (green), nuclei were visualized by DAPI staining (blue). Merging the signals depicts the missing colocalization of MCPH1 with centrosomes (lower panel), a detailed view of the centrosomes is shown in the upper right corner. Scale bar = 10 µm. (TIF) [file pone.0040387.s002.tif]
