# Supplementary material for: A Novel MCPH1 Isoform Complements the Defective Chromosome Condensation of Human MCPH1-Deficient Cells
Source: PLoS One. 2012 Aug 30;7(8):e40387. doi: 10.1371/journal.pone.0040387 (PMC3431399; doi:10.1371/journal.pone.0040387)
Supplement: Table S1 — Computed scores representing predicted strengths of function of the canonical splice donors in human MCPH1. (DOCX) [file pone.0040387.s003.docx]

**Table S1**

Computed scores representing predicted strengths of function of the canonical splice donors in human *MCPH1*

| **Exon:Intron** | **Splice donor*** | **NNSPLICE**** | **Screen 3.4***** |
| --- | --- | --- | --- |
| **1** | AAG:GTGAGGTA | 0.94 | 15.7 |
| **2** | AAG:GTAAGACA | 1.00 | 17.1 |
| **3** | AAA:GTAAGCAG | 0.98 | 12.6 |
| **4** | AAA:GTAAGTAC | 0.99 | 16.1 |
| **5** | TAG:GTAAGCTA | 1.00 | 17.1 |
| **6** | CCT:GTAAGTAA | 0.94 | 15.7 |
| **7** | CAG:GTAAAATT | 0.90 | 15.8 |
| **8** | GAA:GTATGTGA | 0.69 | 11.1 |
| **9** | AAG:GTCAGTGT | 0.94 | 16.3 |
| **10** | TGA:GTAAGTAC | 0.99 | 15.7 |
| **11** | TGG:GTAAGCCC | 0.99 | 15.0 |
| **12** | CCC:GTAAGTCA | 0.98 | 14.0 |
| **13** | TAG:GTAAGAAT | 1.00 | 18.6 |

* Nucleotide positions -3 through +8 relative to the splice site.

** http://www.fruitfly.org/seq_tools/splice.html

*** http://www.uni-duesseldorf.de/rna/html/hbond_score.php
