# Supplementary material for: A Novel MCPH1 Isoform Complements the Defective Chromosome Condensation of Human MCPH1-Deficient Cells
Source: PLoS One. 2012 Aug 30;7(8):e40387. doi: 10.1371/journal.pone.0040387 (PMC3431399; doi:10.1371/journal.pone.0040387)
Supplement: Table S2 — Expression of MCPH1 splicing variants in human adult and fetal tissues. (DOCX) [file pone.0040387.s004.docx]

**Table S2**

Expression of MCPH1 splicing variants in human adult and fetal tissues*

| **Adult tissues** | **MCPH1-FL** | | **MCPH1Δe9-14** | | **MCPH1Δe8** | |
| --- | --- | --- | --- | --- | --- | --- |
|  | **Copy number/**  **ng cDNA** | **± 1 S.D.** | **Copy number/**  **ng cDNA** | **± 1 S.D.** | **Copy number/**  **ng cDNA** | **± 1 S.D.** |
| **Heart** | 2644 | 336 | 1291 | 106 | 943 | 109 |
| **Brain** | 2090 | 168 | 2169 | 179 | 99 | 50 |
| **Placenta** | 8962 | 719 | 5910 | 593 | 363 | 101 |
| **Lung** | 2481 | 210 | 3041 | 282 | 61 | 36 |
| **Liver** | 3762 | 689 | 3474 | 514 | 110 | 17 |
| **Skeletal muscle** | 6647 | 1240 | 2461 | 361 | 150 | 34 |
| **Kidney** | 1565 | 177 | 1044 | 125 | 112 | 65 |
| **Pancreas** | 8862 | 717 | 6215 | 805 | 367 | 30 |
| **Spleen** | 7038 | 503 | 4241 | 394 | 112 | 49 |
| **Thymus** | 4160 | 241 | 4254 | 344 | 56 | 24 |
| **Prostate** | 4661 | 460 | 5191 | 545 | 129 | 67 |
| **Testis** | 9639 | 1192 | 10988 | 708 | 370 | 107 |
| **Ovary** | 6105 | 478 | 8390 | 560 | 54 | 31 |
| **Small Intenstine** | 3919 | 358 | 4074 | 564 | 109 | 53 |
| **Colon** | 3763 | 208 | 3502 | 274 | 146 | 62 |
| **Leukocyte** | 4846 | 1428 | 2021 | 279 | 76 | 13 |
| **Fetal tissues** |  |  |  |  |  |  |
| **Brain** | 5799 | 503 | 10568 | 815 | 219 | 113 |
| **Lung** | 2185 | 224 | 3858 | 586 | 126 | 47 |
| **Liver** | 2882 | 396 | 3515 | 414 | 157 | 71 |
| **Kidney** | 4536 | 510 | 4054 | 419 | 145 | 94 |
| **Heart** | 4959 | 678 | 6401 | 994 | 181 | 21 |
| **Spleen** | 2367 | 280 | 3457 | 508 | 81 | 10 |
| **Thymus** | 9294 | 1594 | 9067 | 1488 | 132 | 63 |
| **Skeletal muscle** | 3843 | 1538 | 5078 | 2340 | 131 | 24 |

* Data shown represent means ± 1 S.D. of three different experiments and are normalized to the geometric average expression levels of *UBC*, *GAPDH*, *B2M*, and *HPRT1*.
